# Histone Deacetylase Inhibitors Increase p27^Kip1^ by Affecting Its Ubiquitin-Dependent Degradation through Skp2 Downregulation

**DOI:** 10.1155/2016/2481865

**Published:** 2015-11-22

**Authors:** Adriana Borriello, Silvio Naviglio, Debora Bencivenga, Ilaria Caldarelli, Annunziata Tramontano, Maria Carmela Speranza, Emanuela Stampone, Luigi Sapio, Aide Negri, Adriana Oliva, Antonio Agostino Sinisi, Annamaria Spina, Fulvio Della Ragione

**Affiliations:** ^1^Department of Biochemistry, Biophysics and General Pathology, Second University of Naples, 80138 Naples, Italy; ^2^Dipartimento di Scienze Biomediche Sperimentali e Cliniche, Università degli Studi di Firenze, 50134 Firenze, Italy; ^3^Dipartimento di Scienze Cardiotoraciche e Respiratorie, Seconda Università di Napoli, 80131 Napoli, Italy

## Abstract

Histone deacetylase inhibitors (HDACIs) represent an intriguing class of pharmacologically active compounds. Currently, some HDACIs are FDA approved for cancer therapy and many others are in clinical trials, showing important clinical activities at well tolerated doses. HDACIs also interfere with the aging process and are involved in the control of inflammation and oxidative stress.* In vitro*, HDACIs induce different cellular responses including growth arrest, differentiation, and apoptosis. Here, we evaluated the effects of HDACIs on p27^Kip1^, a key cyclin-dependent kinase inhibitor (CKI). We observed that HDACI-dependent antiproliferative activity is associated with p27^Kip1^ accumulation due to a reduced protein degradation. p27^Kip1^ removal requires a preliminary ubiquitination step due to the Skp2-SCF E3 ligase complex. We demonstrated that HDACIs increase p27^Kip1^ stability through downregulation of Skp2 protein levels. Skp2 decline is only partially due to a reduced Skp2 gene expression. Conversely, the protein decrease is more profound and enduring compared to the changes of Skp2 transcript. This argues for HDACIs effects on Skp2 protein posttranslational modifications and/or on its removal. In summary, we demonstrate that HDACIs increase p27^Kip1^ by hampering its nuclear ubiquitination/degradation. The findings might be of relevance in the phenotypic effects of these compounds, including their anticancer and aging-modulating activities.

## 1. Introduction

The remodelling of nucleosome is a key epigenetic mechanism for modulating genome expression. Particularly, it has been postulated that histones postsynthetic modifications represent a “code” that can be recognized by nonhistone proteins, resulting in dynamic complexes capable of regulating gene transcription. Several reversible posttranslational changes of histone proteins have been described including lysine acetylation, lysine and arginine methylation, serine phosphorylation, proline isomerization, lysine ubiquitination and sumoylation, ADP-ribosylation, deimination, crotonylation, propionylation, butyrylation, formylation, hydroxylation, and* O*-GlcNacylation [1, 2].

Two groups of enzymes, histone acetyltransferases and histone deacetylases (HDACs), determine the pattern of histone acetylation. In humans, there are 18 identified HDACs, classified into four major classes, on the basis of sequence homology [3, and references therein]. Class I HDACs (HDAC1, HDAC2, HDAC3, and HDAC8) are strictly related to the yeast transcriptional regulator RPD3 [2], while class II HDACs (HDAC4, HDAC5, HDAC6, HDAC7, HDAC9, and HDAC10) share domains with similarity to HDA1, another deacetylase found in yeast. Then, a new member of the HDAC family (HDAC11) has been identified. So far, it represents the only member of class IV HDAC enzymes [3, 4]. Class I, II, and IV HDACs constitute the “classical” HDAC family, with a common catalytic mechanism that requires a zinc metal but does not involve a cofactor. Finally, the sirtuin family (sirtuins 1–7) belongs to class III HDACs, which share homology with the yeast SIR2 protein [5]. These enzymes employ a distinct catalytic mechanism, which is NAD^+^-dependent.

HDACs of class I are almost ubiquitously expressed, whereas the distribution of class II HDACs is more specific, these enzymes being strongly involved in the development and function of skeletal muscle and brain and in the immunity system (innate immunity) [4]. The functions and tissue distribution of class III HDACs (the sirtuin family in high eukaryotes) still require further evaluation. There is however ample evidence that these enzymes are involved in the ageing process, in the response to the caloric restriction, and in many metabolic processes [5].

The reprogramming of the physiological histone acetylation process by environmental alterations or drug treatments generally results in marked phenotypic changes. This is clearly illustrated by the antiproliferative and anticancer effects of HDAC inhibitors (HDACIs), which have been demonstrated both in cellular and animal models and in clinical trials [6, 7].

HDACIs promote acetylation of histone proteins, allowing the transcriptional machinery to access DNA and modulating/enhancing gene transcription, and also of nonhistone protein substrates, such as transcription factors, DNA-binding proteins, DNA-repair proteins, signal-transduction factors, and chaperone proteins. The nonhistone proteins modifications are then able to interfere with many fundamental processes, including gene expression, mRNA half-life, protein stability and activity, and reactive oxygen species generation [4].

The first HDACI extensively investigated is the short chain fatty acid butyric acid (BuA). The molecule and its derivatives (particularly phenylbutyrate, PBuA) induce, in all the cell lines investigated, important morphological and phenotypic changes which vary from cell to cell and include proliferation arrest, differentiation, and apoptosis [4, 8–10]. BuA has also been found to have potentially antineoplastic effects on colonic mucosal epithelium [9]. Due to its antiproliferative activity, BuA has been actively investigated as a potential anticancer agent [4]. The molecule has also shown strong anti-inflammatory and antioxidant effects in several disease models such as ulcerative colitis, type 1 diabetes, or atherosclerosis [4, 11]. In particular, BuA exhibits an antioxidant effect by augmenting cellular glutathione, reducing reactive oxygen species levels, and upregulating several glutathione-S-transferase isoforms, implicating the activation of an antiredox component in the antiproliferative effect of BuA. Moreover, the absence of toxicity and the capability of reactivating gene expression have compelled numerous studies on BuA therapeutic applications.

Therefore, BuA and its derivatives have been evaluated in human trials [12, 13] for reexpression of silenced globin genes as a therapy for sickle cell anemia [14] and *β*-thalassemia [14, 15]. In addition, the molecule has been in clinical testing for targeted antitumor therapies, and data on its use in the treatment of virus-dependent malignancies (namely, Epstein-Barr virus-derived lymphomas) have been so far encouraging [16]. The utilization of the molecule in therapy is however hampered by its extremely short* in vivo* half-life [4].

This, in turn, stimulated the synthesis of more efficacious and stable HDACIs. The novel HDACIs reported to date might be classified into several structural classes, including hydroxamates, cyclic peptides, aliphatic acids, and aminobenzamides [4, 7]. Trichostatin A was the first natural hydroxamate discovered to inhibit HDACs. Vorinostat (suberanilohydroxamic acid, SAHA) is structurally similar to trichostatin A and a nanomolar inhibitor of partially purified HDAC classes I and II [17]; to date, it has been FDA approved, together with romidepsin, for the treatment of refractory cutaneous T cell lymphoma (CTCL) and is under clinical trials for therapy of several other malignancies. Additional potent HDACIs include M-carboxycinnamic acid bishydroxamide and its derivative, MS-275, apicidin, and others [7]. Their use in cancer treatment is under evaluation in clinical trials [4]. In addition, valproic acid (VPA), a drug employed in the therapy of epilepsy and bipolar syndrome, inhibits HDACs of types I and II [18], suggesting its use as a chemotherapeutic agent, too.

Generally, HDACIs cause the arrest of cell proliferation during the transition from G1 to S phase, causing the accumulation of cells in G1. This event is followed by differentiation or apoptosis, mostly depending on (i) the molecule and its concentration, (ii) the treatment duration, and (iii) the cell model or, in other words, the genetic lesions present in cancer cells.

Cyclin-dependent kinase (CDK) inhibitors (CKIs) act on various cyclin-CDK complexes during different phases of the cell cycle. Particularly, CKIs, such as p21^Cip1^, p27^Kip1^, and p57^Kip2^, have been shown to mediate the G1 arrest in response to an array of stimuli including DNA damage, mitogen deprivation, or drug treatments [19–21].

Here, we report a study on the effect of HDACIs on p27^Kip1^, a tight-binding inhibitor of CDK complexes, belonging to the Cip/Kip family. An extensive literature demonstrates that the protein is significantly involved in the modulation of several different cellular processes, including proliferation, differentiation, apoptosis, cell movement, and metastasization [20, 21].

We demonstrated that the antiproliferative effect of class I/II HDAC inhibitors is associated with a strong increase of the CKI and that this event is due to the elongation of p27^Kip1^ half-life. A bidimensional analysis of p27^Kip1^ shows that the protein accumulates predominantly in a monophosphorylated isoform. Intriguingly, the impairment of the CKI removal is associated with the HDACI-dependent decrease of Skp2, a component of the ubiquitination machinery of p27^Kip1^.

## 2. Materials and Methods

### 2.1. Materials and Antibodies

BuA, PBuA, VPA, nicotinamide (NAM), cycloheximide, and the cysteine protease inhibitor E64 were supplied by Sigma Chemical Company, St. Louis, MO, USA. The proteasome inhibitors N-acetyl-leucyl-leucyl-norleucinal (ALLN) and* clasto*-lactacystin *β*-lactone (CLBL) were furnished by BioMol Research Laboratories Inc., PA, USA. MS-275 was from Calbiochem EMD Biosciences, Inc., La Jolla, CA, USA. Splitomicin was supplied by Alexis Biochemicals, San Diego, CA, USA. TurboFect Transfection Reagent was purchased from Thermo Fisher Scientific Inc. (Waltham, MA, USA). The cell culture media and supplements were purchased from Invitrogen s.r.l. (Invitrogen, Carlsbad, CA). pcDNA3-Skp2 and pcDNA3.0-ubiquitin-cMyc were kind gifts of Dr. D. Germain (Department of Medicine, Division of Hematology/Oncology, Mount Sinai School of Medicine, New York, USA). Validated siRNAs directed against Skp2 (catalog numbers 1471, 1565, and 1655) were furnished by Ambion (Europe) Ltd., Huntingdon, Cambridge, UK.

Monoclonal antibodies (MAb) to p27^Kip1^ were from BD Transduction Laboratories (Franklin Lakes, NJ, USA). Polyclonal antibodies (PAb) against Skp2 (H-435), p27^Kip1^ (C19), p57^Kip2^ (sc-8298), p21^Cip1^ (sc-397), CDK2 (M2), phosphoserine 10-p27^Kip1^ (sc-12939-R), phosphothreonine 187-p27^Kip1^ (sc-16324), phospho-GSK3*β* (sc-11757), acetyl-histone H3 (sc-8655) and MAb to PARP [poly(ADP-ribose)polymerase] (sc-8007), GSK3*β* (sc-377213), *β*-tubulin (sc-5274), and cMyc (sc-40) were from Santa Cruz Biotechnologies (Santa Cruz, CA, USA). PAb against actin and histone H3 were from Sigma. PAb against acetyl-histone H3 were from Millipore Corporation (Upstate) (Billerica, MA, USA).

### 2.2. Cell Lines, Treatments, and Cellular Fractionation

The source of the cell lines employed and their culture conditions were previously reported [22–24]. In particular the following cell lines were employed: Lan-5, SK-N-SH, and SK-N-BE (all from human neuroblastoma), RD (from a human rhabdomyosarcoma), HeLa (from a human cervical cancer), EPN (from human prostate epithelium), K562 and JURL-MK1 (from human chronic myelogenous leukemias), Caco2 (from a colon cancer), and UT-7 (from a megakaryoblastic leukemia). To avoid effects due to the cell-to-cell contact inhibition, all the treatments were performed on cultures kept at low density (3-4 × 10^5^ cells/mL in 100 mm dishes, for cells growing in suspension).

### 2.3. Flow Cytometry and Western Blotting Analyses

Cell cycle distribution was studied by propidium iodide staining and flow cytometric (FACS) analysis using a FACScalibur (Becton Dickinson, CA, USA) as described in [25]. The obtained results were calculated from 40.000–60.000 events with ModFit LTTM software (Becton Dickinson, CA, USA). Total cellular extracts were prepared as in [26]. Nuclear and cytosol extracts were obtained using NE-PER Nuclear and Cytoplasmic Extraction Reagents (Pierce Biotechnology, Rockford, IL, USA) and analyzed by western blotting as previously described [23, 24]. Immunoblotting signals were quantified by the analysis of scanner band intensities using ImageJ64 software. To determine the effect of BuA on p27^Kip1^ half-life, control and 16-hour BuA-treated cells were grown in presence of the protein synthesis inhibitor cycloheximide at 36 *μ*M final concentration; then samples were withdrawn at different time points and analyzed by immunoblotting.

### 2.4. Two-Dimensional Electrophoresis Analyses

Two-dimensional gel electrophoresis separations were performed as in [26]. For the first dimension Immobiline DryStrip gels, linear pH (3–10 or 4–7), were employed. After isoelectrofocusing, the strips were equilibrated in 50 mM Tris (pH 8.5), 6 M urea, 30% glycerol, 2% SDS, 1% DTT, and 0.002% bromophenol blue and loaded onto SDS-polyacrylamide gels for separation according to molecular mass. After transfer on nitrocellulose membranes, the proteins were immunodetected by means of the relative specific antiserum.

### 2.5. Reverse-Transcriptase Polymerase Chain Reaction (RT-PCR) and Quantitative Polymerase Chain Reaction (qPCR)

Total RNA was extracted, purified, and retrotranscribed as previously described [23]. RT-PCR conditions and primer sequences for glyceraldehyde 3-phosphate dehydrogenase (GAPDH), p27^Kip1^, and Skp2 will be available upon request. In some experiments, the RT-PCR analysis was performed on cells incubated in presence of 36 *μ*M cycloheximide. qPCR analyses were performed for evaluating the expression of* Skp2*. The PCR conditions were described in [23] and the sequences of the employed primers are available on request.

### 2.6. Plasmid Transfection

pcDNA3-Skp2 and pcDNA3.0-ubiquitin-cMyc were transiently transfected into EPN using TurboFect Transfection Reagent (Thermo Fisher Scientific) following the manufacturer's instructions. Briefly, EPN cells, plated in a 6-well tissue culture plate, were transfected with the selected pcDNA3 (3,0 *μ*g DNA/500,000 cells). At the end of incubation, the cells were washed with fresh medium and treated as reported in the text. Extracts of the transfected cells were analyzed by immunoblotting. The treatment with siRNA against Skp2 was performed as follows. 24 hours before siRNA addition, EPN cells were seeded in 6-well tissue culture plates and cultured in DMEM/F12-HAM (Sigma) supplemented with 5% FBS without antibiotics. Transfection was performed by Lipofectamine 2000 following the manufacturer's instructions and using 40 nM siRNA final concentration. After 48 hours, cells were analyzed for Skp2 content by immunoblotting. CDK2 assay was performed essentially as described in [26].

### 2.7. Statistical Analysis

Experimental data are expressed as mean ± S.D. GraphPad Prism 6 (GraphPad Software, La Jolla, CA, USA) was used for statistical analysis. Comparisons between treated samples and control were performed using sample *t*-test or ANOVA. *P* value < 0.05 was considered to be significantly different.

## 3. Results

### 3.1. The Antiproliferative HDACIs Effect Is Associated with p27^Kip1^ Accumulation

**Figure 1:**
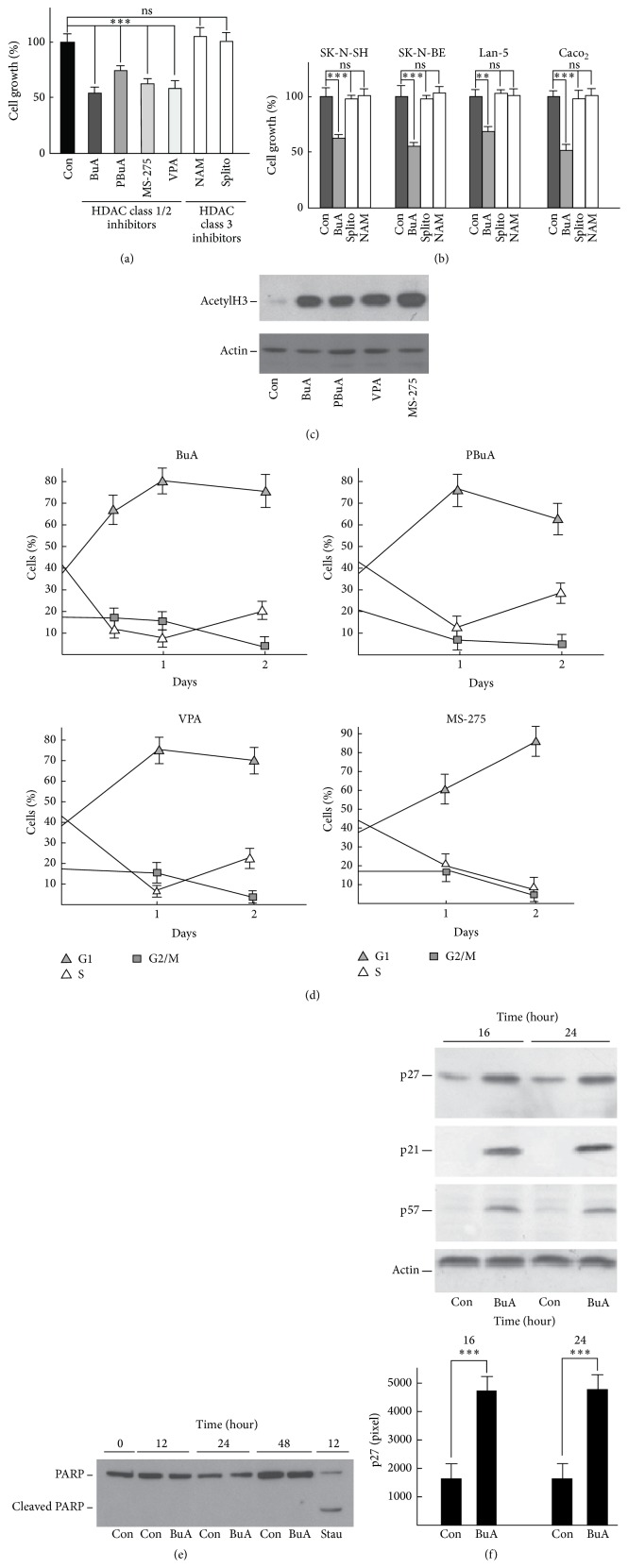
Effect of inhibitors of HDACs on cellular growth and phenotype. (a) K562 cells were plated at 300.000 cells/mL and incubated without (Con) and with 1 mM butyric acid (BuA), 2 mM phenylbutyric acid (PBuA), MS-275 (1 *μ*M), 2 mM valproic acid (VPA), 100 *μ*M splitomicin (Splito), and 10 mM nicotinamide (NAM). The effect on the growth is expressed as percentage of control after 2 days of incubation. The reported data represent the mean values ± S.D. of three independent experiments, with each experiment performed in duplicate. Con, untreated cells; ^*∗∗∗*^ *P* < 0.001 compared to Con; ns, not significant compared to Con. (b) Various cell lines were incubated without (Con) and with BuA, Splito, and NAM at the concentrations reported in (a). The effect on the growth is expressed as percentage of control after 2 days of incubation. The reported data represent the mean values ± S.D. of three independent experiments, with each experiment performed in duplicate. Con, untreated cells; ^*∗∗∗*^ *P* < 0.001 compared to Con; ns, not significant compared to Con. (c) K562 cells were plated and treated as in (a). Cell extracts were prepared as in Materials and Methods and analyzed by immunoblotting for the content of acetyl-histone H3 (AcetylH3). Actin was evaluated for confirming equal protein loading. (d) The panel reports the flow cytometric analyses of K562 cells treated with BuA, PBuA, VPA, and MS-275 (at concentrations reported in (a)) for different time intervals. At each time (24 and 48 hours) the percentage of cells in a specific phase is showed. Time 0 represents the percentage of specific phase of untreated growing cells. The reported data represent the mean values of three independent experiments. The percentage of subG1 cells is lower than 2% in all the FACS analyses. (e) K562 cells were incubated without (Con) and with BuA (1 mM) for the reported time intervals. Total cell extracts were then prepared as in Materials and Methods and analyzed by immunoblotting for PARP and PARP cleavage product (as marker of apoptosis). 1 *μ*M staurosporine-treated cell extract was used as a positive control. (f) K562 cells were incubated without (Con) and with BuA (1 mM) for 16 and 24 hours. Total cell extracts were prepared as in Materials and Methods and analyzed by immunoblotting for p27^Kip1^ (p27), p21^Cip1^ (p21), and p57^Kip2^ (p57). For the densitometric quantitation of p27^Kip1^ analysis, three identical experiments were performed and the relative films were scanned and analyzed by ImageJ64 software. The data reported (as pixel) represent the mean values ± S.D. ^*∗∗∗*^ *P* < 0.001 compared to untreated cells (Con).

When K562 cells were treated with different class 1 and 2 HDAC inhibitors (BuA, PBuA, VPA, and MS-275), a clear and statistical significant inhibition of growth occurred. The effect was evaluated by direct cell counting and was expressed as percentage of cells with respect to control (i.e., untreated cells) (Figure 1(a)). Conversely, when these cells were incubated with two powerful inhibitors of sirtuin proteins (class 3 HDACs), that is, NAM and splitomicin, no effect on the growth rate was observed (Figure 1(a)). The finding was confirmed by using the additional cell lines reported in Figure 1(b). Identical results were obtained with other cell lines, that is, RD, HeLa, and EPN cells (data not shown).  Figure 1(c) confirms the effects of the molecules on histone acetylation.

Flow cytometry analyses of K562 cells grown in the presence of HDACIs are reported in Figure 1(d). The FACS data obtained showed that the addition of BuA, PBuA, and VPA induces, during the first 24 hours, a rapid accumulation of cells in G1 phase along with a decrease of cells in S phase. The G2/M cell percentages were almost unchanged. At 48 hours, the patterns changed (equally for BuA, PBuA, and VPA), in that the number of cells in S phase statistically increased while G2/M cells and G1 cells decreased. These findings argued in favour of inhibitory effects, acting at different time windows, on two restriction points (i.e., G1 → S and S → G2). The effect of MS-275 on cell division cycle appears distinct: the cell percentage in G1 phase progressively augmented while those in S and G2/M phases gradually diminished (Figure 1(d)). We did not evidence accumulation of cells in subG1 suggesting the absence of apoptosis in all the treatments. The absence of cell programmed death was confirmed by the evaluation of PARP cleavage, a marker of apoptosis. Up to 48 hours of BuA treatment, no cleavage of the protein was observed (Figure 1(f)). Conversely, a brief treatment of K562 cells with staurosporine (an apoptotic activator) caused the appearance of a significant PARP cleavage product (Figure 1(e)).

Since the HDACIs induced, at least during the first 24 hours of treatment, the accumulation of cells in G1 phase, we investigated the levels of the Cip/Kip family protein members p21^Cip1^, p27^Kip1^, and p57^Kip2^, CKIs involved in the control of the G1 → S phase transition.

As reported in Figure 1(e), all the CKIs levels were increased by BuA treatment in K562 cells. However, while the results on p21^Cip1^ and p57^Kip2^ confirmed several previous observations and, thus, represent well-consolidated findings ([27, 28] and references therein), the data regarding p27^Kip1^ appear most appealing, since scarce knowledge on the activity of HDACIs on this protein is still available [29–31].

**Figure 2:**
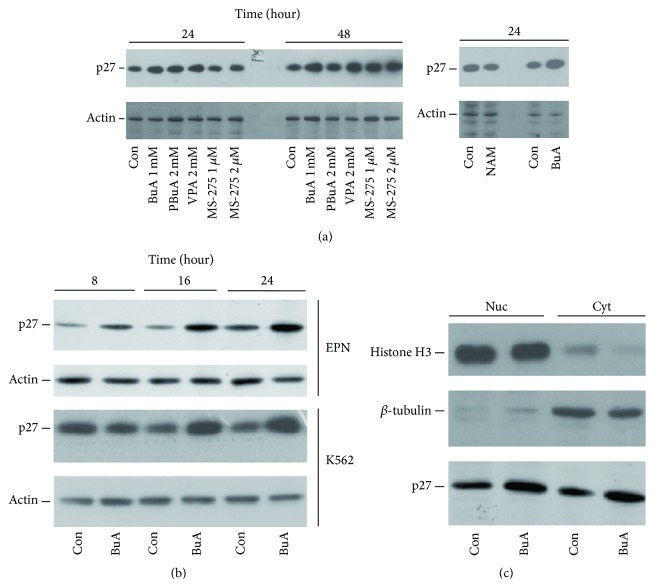
Effect of HDACIs on the p27^Kip1^ cellular content. (a)* On the left*, K562 cells were incubated with different HDACIs for 24 and 48 hours at the concentrations showed. Then, total cell extracts were prepared and the content of p27^Kip1^ was determined.* On the right*, K562 cells were incubated with NAM and BuA at the concentration reported in Figure 1(a) for 24 hours. Then, total cell extracts were prepared and the content of p27^Kip1^ was determined. Actin was also analyzed for confirming equal loading. (b) EPN and K562 cells were incubated for different time periods with 1 mM BuA. Subsequently, p27^Kip1^ and actin contents were determined by immunoblotting. Actin was used for verifying equal loading. (c) K562 cells were incubated for 16 hours with 1 mM BuA. Then, the nuclear and cytosolic compartments were prepared. Finally, the content of p27^Kip1^ and HDAC1 was determined by immunoblotting. Histone H3 and *β*-tubulin were used as markers of nuclear and cytosolic fraction, respectively, and for equal loading evaluation.

We strengthened the view that the BuA effect on p27^Kip1^ was related to HDAC inhibition by employing PBuA, VPA, and MS-275 (Figure 2(a), left). Interestingly, NAM did not induce p27^Kip1^ increase (Figure 2(a), right). Moreover, we confirmed the data obtained in K562 cells by using other cell systems, including EPN, HeLa, HT29, and Caco2 cell lines and EBV-transformed lymphoblastoid cells (data not shown). In particular, EPN, which is a cell line established from human prostate epithelium, when treated with BuA showed a very strong effect on the CKI content. Thus, this cell line, together with K562 cells, was employed in the majority of the subsequent experiments. When time-course analyses were performed in EPN and K562 cells to evaluate the effect of BuA treatment on p27^Kip1^ cellular content, we observed that the activity of HDACIs was clearly detected after 16 hours of incubation (Figure 2(b)).

Thereafter, we investigated if BuA causes the accumulation of p27^Kip1^ in a specific cellular compartment. Indeed, it has been demonstrated that the relocalization of the CKI plays a pivotal role in modulating its metabolism and activity ([20, 21] and references therein). However, as shown in Figure 2(c), after 16 hours of treatment, the build-up of the protein was observed in both nuclei and cytosols of the treated cells.

### 3.2. The HDACI-Dependent p27^Kip1^ Increase Is due to Reduced Protein Degradation

**Figure 3:**
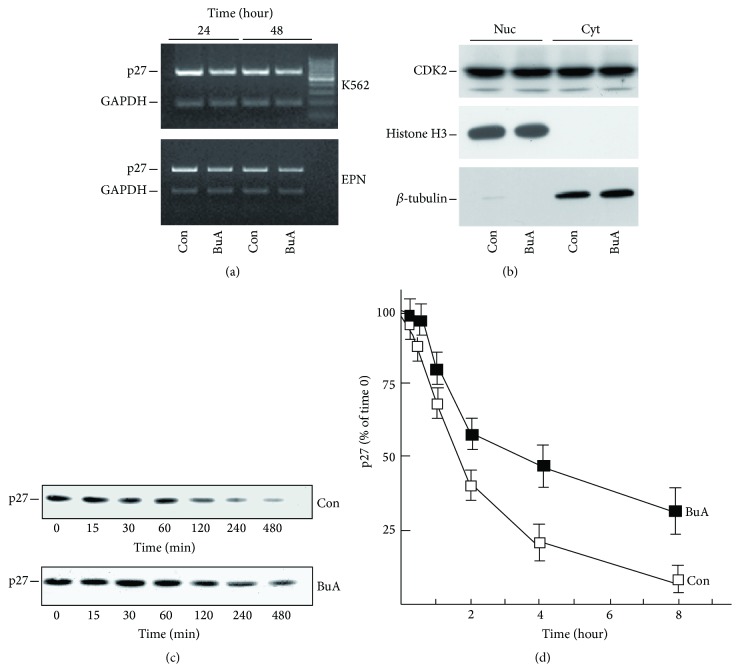
Effect of BuA on the expression and turnover of p27^Kip1^. (a) K562 and EPN cells were treated with BuA 1 mM for different time intervals and then total RNA was prepared. Subsequently, the contents of p27^Kip1^ (p27) and glyceraldehyde 3-phosphate dehydrogenase (GAPDH) transcripts were determined by RT-PCR (see Materials and Methods for experimental details). GAPDH was used as internal standard. (b) K562 cells were incubated for 24 hours with 1 mM BuA. Then, the nuclear and cytosolic compartments were prepared and content of CDK2 was determined by immunoblotting. Histone H3 and *β*-tubulin were used as markers of nuclear and cytosolic fraction, respectively, and for equal loading evaluation. (c) K562 cells were incubated without (Con) and with 1 mM BuA 1 for 16 hours. Then, the cells were pelleted and washed for removing BuA and 36 *μ*M cycloheximide was added to the culture medium. Finally, cells were collected at different time intervals and analyzed for the content of p27^Kip1^ (p27) by immunoblotting. (d) Three independent experiments as those reported in (c) were performed. The results of blots were analyzed and quantified by ImageJ64 software. The graph reports the average for each time period of these experiments and the bar the statistical variation.

The analysis of BuA activity on the expression of p27^Kip1^ gene (*CDKN1B*) demonstrated, both in K562 and in EPN cells, that the compound does not upregulate* CDKN1B* transcription (Figure 3(a)). Since it has also been reported that the cytosolic relocalization of CDK2, a key CDK modulated by p27^Kip1^, causes the increase of the CKI [32], we also investigated the effect of BuA on the cellular distribution of the kinase. No changes in the CDK2 localization were observed after 24 hours of treatment, allowing the exclusion of this mechanism as the cause of p27^Kip1^ accumulation (Figure 3(b)). We subsequently hypothesized that HDACI-dependent p27^Kip1^ increase might be due to an effect on the CKI degradation. Accordingly, control and 16-hour BuA-treated K562 cells were incubated with cycloheximide and, then, the cells were collected at various time intervals. As shown in Figures 3(c) and 3(d), a clear elongation of p27^Kip1^ half-life was observed in cells grown in presence of BuA. Indeed, while the CKI half-life was about 1.5 ± 0.2 hours in untreated K562 cells, it became about 3 ± 0.3 hours in treated cells. The finding suggests that the addition of BuA to the cells reduces their capability of degrading p27^Kip1^.

### 3.3. HDACIs Strongly Downregulate Skp2 Protein, a Component of the Nuclear p27^Kip1^ Ubiquitination Complex

**Figure 4:**
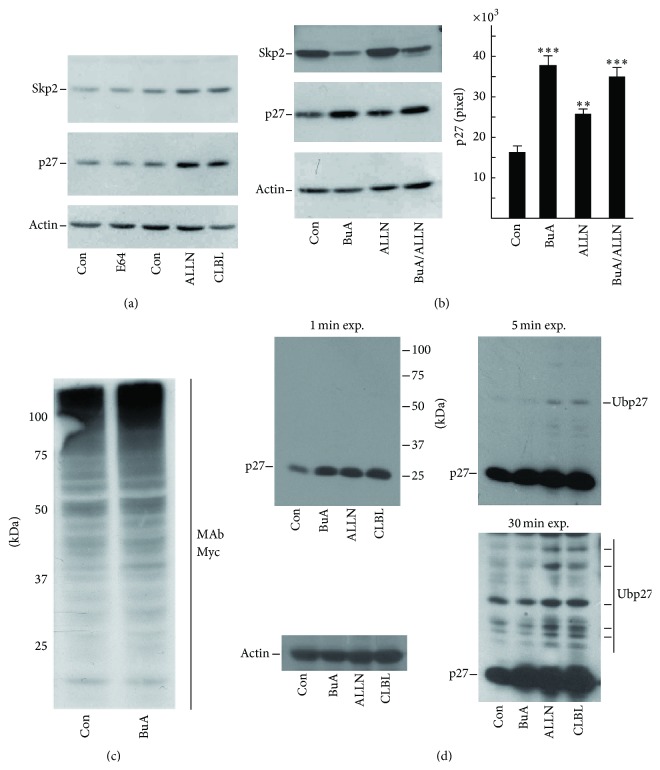
Effect of BuA on the proteasome degradation of p27^Kip1^. (a) EPN cells were treated for 8 hours with different inhibitors of proteolytic activities, that is, E64 (inhibitor of cysteine protease, 100 *μ*M) and ALLN (N-acetyl-leucyl-leucyl-norleucinal, 50 *μ*M) and CLBL (*clasto*-lactacystin *β*-lactone, 50 *μ*M). After 8 hours of incubation, cells were collected and the contents of p27^Kip1^ (p27) and Skp2 were determined by immunoblotting. Actin was analyzed for confirming equal loading. (b)* On the left*, EPN cells were treated with BuA for 24 hours or ALLN for 8 hours or BuA (24 hours) plus LLnL (the final 8 hours). Then, the abundance of p27^Kip1^ (p27), Skp2, and actin was determined by immunoblotting.* On the right*, for the densitometric quantitation of p27^Kip1^ analysis, three identical experiments were performed and the relative films were scanned and analyzed by ImageJ64 software. The data reported (as pixel) represent the mean values ± S.D. ^*∗∗*^ *P* < 0.005 and ^*∗∗∗*^ *P* < 0.001 compared to untreated cells (Con). (c) EPN cells were transfected for 24 hours with pcDNA3.0-ubiquitin-cMyc. Then, the cells were treated or not with BuA for 24 hours. Finally, cell extracts were analyzed by immunoblotting employing a monoclonal antiserum (MAb) directed against cMyc. On the right of blot are reported the molecular weight standard values (kDa). (d) K562 cells were treated (or not) with BuA for 24 hours or with ALLN or CLBL for 12 hours. Then, cellular extracts were prepared and analyzed for p27^Kip1^ and actin content. The blots of p27^Kip1^ were exposed for different times (as shown) in order to show the ubiquitination p27^Kip1^ derivatives. On the top blot (right) are also reported the molecular weight standard values (kDa).

It is definitely known that p27^Kip1^ is committed to removal by ubiquitination followed by proteasomal degradation. Accordingly, several studies demonstrated that proteasome inhibitors increase p27^Kip1^ half-life in different cell systems. This was confirmed by us in EPN cells: we observed that the addition of ALLN and CLBL, two powerful and specific inhibitors of proteasome activity, increased p27^Kip1^ cellular content, while E64 (a cysteine protease inhibitor) did not affect the CKI level (Figure 4(a)). We also evaluated the effect of the protease inhibitors on the levels of Skp2, a protein playing a pivotal role in p27^Kip1^ ubiquitination (and thus removal). As already reported in literature [33, 34], a build-up of Skp2 levels was detected in proteasome inhibitors treated cells confirming that also Skp2 abundance is modulated by ubiquitination (due to APC/Cdh1 complex) and proteasomal degradation (Figure 4(a)).

Then, we compared the effect of BuA with that of ALLN on p27^Kip1^ cellular content. As shown in Figure 4(b), both ALLN and BuA caused a remarkable increase of p27^Kip1^ levels. Importantly, the contemporaneous addition of the two molecules resulted in a faint additive effect on p27^Kip1^ content, suggesting that both ALLN and BuA act on the same pathway. When we evaluated the activity of the treatments on Skp2 levels, we observed that the HDACI strongly reduces Skp2 cellular content. This reduction was partially prevented by the addition of ALLN, probably due to its capability of hampering Skp2 proteasomal degradation (Figure 4(b)).

The strong negative effect of BuA on Skp2 levels represents a promising observation since it might explain (at least in part) the HDACI-dependent p27^Kip1^ increase. A putative biochemical consequence of this finding is that BuA addition should allow the accumulation of p27^Kip1^ but not of its polyubiquitinated derivatives, while the addition of proteasome inhibitors (ALLN or CLBL) should increase both unmodified p27^Kip1^ and its polyubiquitinated isoforms. We also considered the possibility that BuA might affect the* in toto* cellular ubiquitination process and not only the mechanisms controlled by specific E3 ligase ubiquitinating complexes (like the Skp2-SCF E3 ubiquitin ligase). To explore the last hypothesis, we transfected EPN cells with an expression vector codifying for Myc-tagged ubiquitin and evaluated the degree of total protein ubiquitination by employing monoclonal antibodies against Myc. BuA did not significantly modify, either quantitatively or qualitatively, the whole cell protein ubiquitination pattern (Figure 4(c)).

Subsequently, we investigated the effect of HDACI-dependent Skp2 decrease on p27^Kip1^ ubiquitination. As showed in Figure 4(d), we induced a build-up of p27^Kip1^ by different treatments (BuA, ALLN, and CLBL), obtaining a strong CKI upregulation. Importantly, the levels of p27^Kip1^ were similar in all the treated samples (Figure 4(d)). However, no increase of ubiquitinated p27^Kip1^ bands was observed in BuA-treated cells with respect to the control. Conversely, proteasome inhibitors caused a significant accumulation of ubiquitinated p27^Kip1^ isoforms. Different expositions of the blot were reported to clearly evidence differences in p27^Kip1^ polyubiquitination patterns (Figure 4(d)).

**Figure 5:**
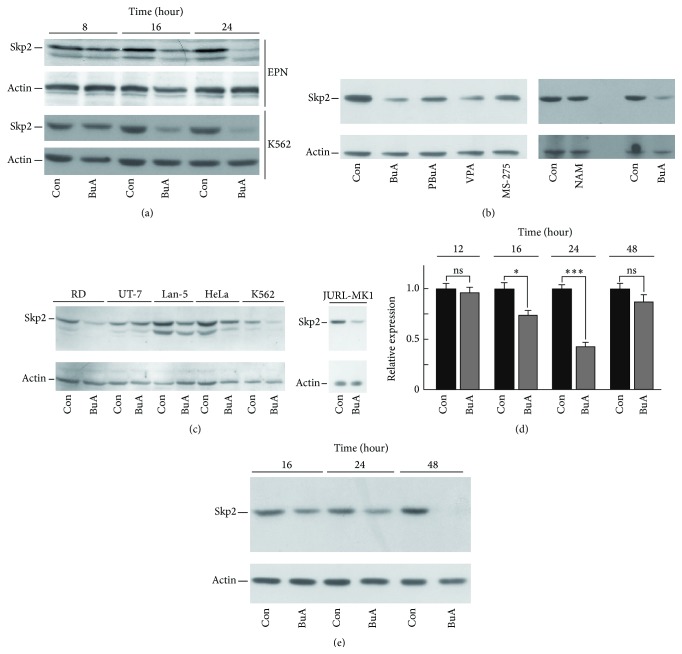
Effect of HDACIs on Skp2 content. (a) EPN and K562 cells were treated with 1 mM BuA for the indicated time periods. Then, cellular extracts were prepared and Skp2 was determined by immunoblotting. Actin was evaluated for confirming equal loading. Con, untreated cells. (b)* On the left*, K562 cells were incubated for 24 hours with different HDACI at the concentrations indicated in Figure 1(a). Then, cellular extracts were prepared and analyzed for Skp2 and actin content by immunoblotting.* On the right*, K562 cells were cultured in the presence of BuA and NAM (as in Figure 1(a)). Then, cellular extracts were prepared and analyzed for Skp2 and actin content by immunoblotting. Con, untreated cells. (c) Various cell lines (RM, UT-7, Lan-5, HeLa, K562, and JURL-MK1) were cultured with 1 mM BuA for 24 hours. Then, Skp2 and actin were determined by immunoblotting. Con, untreated cells. (d) K562 cells were incubated with BuA for different time periods (as reported). Then, total RNA was prepared, retrotranscribed, and employed for evaluating Skp2 mRNA by quantitative PCR. The experiment was performed in triplicate. Con, untreated cells. ^*∗∗*^ *P* < 0.005 and ^*∗∗∗*^ *P* < 0.001 compared to Con. ns, not significative with respect to Con. (e) K562 cells were treated with 1 mM BuA for 16, 24, and 48 hours. Then, extracts were prepared and Skp2 was determined by immunoblotting. Actin was evaluated for confirming equal loading. Con, untreated cells.

Since Skp2 reduction might explain, at least in part, the HDACI-dependent p27^Kip1^ accumulation, we investigated this effect in detail. As shown in Figure 5(a), BuA strongly decreased Skp2 levels in K562 and EPN cells. The effect was evident after 16 hours of treatment and Skp2 was scarcely detectable after 24 hours. Additional immunoblotting experiments confirmed that Skp2 was downregulated in K562 cells by all the employed HDACIs (Figure 5(b)). Moreover, BuA activity on Skp2 was observed in several other cellular systems tested, including RD, Lan-5, HeLa, EPN, and JURL-MK1 (Figure 5(c)) and SK-N-SH, SK-N-BE, and Caco2 cell lines (data not reported). In UT-7, a cell line derived from a megakaryoblastic leukemia, the effect of BuA on Skp2 was, however, not noticeable (Figure 5(c)). We also evaluated the effect of BuA on Skp2 gene transcription by quantitative PCR (Figure 5(d)). We observed a significant decrease only after 24 hours of incubation while, at 48 hours of treatment, the transcript reduction, with respect to untreated cells, was not statistically significant. Conversely, as shown in Figure 5(e), Skp2 protein progressively diminishes in BuA-treated K562 cells starting from 16 hours. At 48 hours of BuA treatment, Skp2 signal was scarcely detectable (or undetectable).

**Figure 6:**
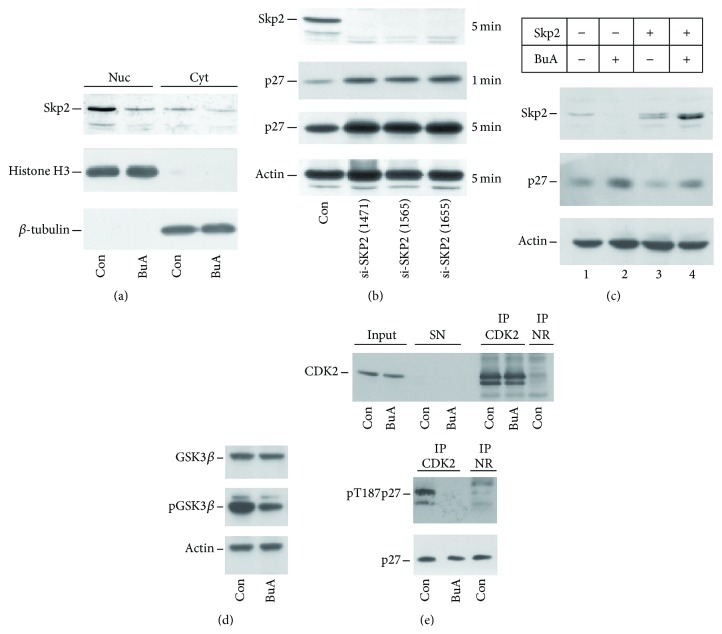
Mechanisms of BuA activity on Skp2 abundance. (a) K562 cells were incubated for 16 hours with 1 mM BuA. Then, the nuclear and cytosolic compartments were prepared. Finally, content of Skp2 was determined by immunoblotting. Histone H3 and *β*-tubulin were used as markers of nuclear and cytosolic fraction, respectively, and for equal loading evaluation. (b) EPN cells were transfected with three different convalidated siRNAs directed against Skp2 mRNA. After 72 hours, the amount of Skp2, p27^Kip1^, and actin content was determined by immunoblotting. In the brackets the catalog number of the siRNA is reported. (c) EPN cells were transfected with pcDNA3-Skp2 plasmid or treated with BuA (or both). After 48 hours, the amount of Skp2 and p27^Kip1^ (p27) content was determined by immunoblotting. Actin was evaluated for confirming equal loading. (d) K562 cells were cultured with or without BuA for 16 hours. Then, cell extracts were prepared and analyzed by immunoblotting for GSK3*β* and phospho-GSK3*β* (pGSK3*β*). Actin was also determined for confirming equal loading. (e) K562 cells were cultured with or without BuA for 16 hours. Then, cell extracts were prepared. CDK2 was immunoprecipitated from control (Con, untreated) and treated cells. On the top is showed CDK2 abundance in the input extracts, CDK2 remaining in the supernatants (to confirm the total precipitation), and CDK2 in 1/10 of IP. An IP not related (NR) is also showed. On the bottom is reported the mixture of assay. CDK2 immunoprecipitated from Con and BuA was employed to phosphorylate recombinant p27^Kip1^ on threonine 187. The mixture of assay was analyzed for the content of p27^Kip1^ and phosphothreonine 187-p27^Kip1^ (pT187p27). The IP not related was also employed as control of phosphorylation.

The levels of Skp2 protein in both the nuclear and the cytosolic compartments in response to BuA treatment were also investigated.  Figure 6(a) shows that Skp2 is mostly localized in the nucleus and that it diminished in this compartment after 16 hours of treatment. No relocalization of the protein was detected. To further confirm the meaning of Skp2 downregulation in the increase of p27^Kip1^, we treated EPN cells with different Skp2 siRNAs. As shown in Figure 6(b), the treated cells showed a complete silencing of Skp2 and a significant upregulation of p27^Kip1^. Finally, we evaluated whether forced expression of Skp2 might cause a decrease of p27^Kip1^ level. As shown in Figure 6(c), the accumulation of Skp2 in EPN cells causes a significant downregulation of p27^Kip1^ level. Moreover, the overexpression of Skp2 reduced the effect of BuA on p27^Kip1^ cellular content. It is to underline that BuA might enhance the activity of expression vectors and this general effect might explain the high Skp2 abundance in cells transfected in the presence of the HDACI. Furthermore, the occurrence of various bands in transfected Skp2 is probably due to different degree of phosphorylation.

Since a remarkable difference between Skp2 transcript decrease and Skp2 protein reduction has been observed after BuA treatment, we decided to take into consideration further mechanisms of Skp2 regulation.

However, because NAM did not change p27^Kip1^ and Skp2 abundance, an effect on Skp2 acetylation was discarded. On the other hand, Skp2 has been reported to be phosphorylated and stabilized by Akt1 and CDK2 [35–37]. Thus, we evaluated whether BuA treatment affected the activity of these two kinases. As shown in Figure 6(d), the treatment with the BuA reduced the amount of phosphorylated GSK3*β*, a key Akt1 substrate, suggesting that the HDACI inhibits the activity of Akt1 and confirming previous data [38, 39]. We also evaluated CDK2 activity after BuA treatment by investigating the capability of equal amount of the kinase to phosphorylate human recombinant p27^Kip1^ on T187 residue. As shown in Figure 6(e), after 16 hours of BuA treatment, the CDK2 kinase activity is strongly reduced.

### 3.4. Characterization of p27^Kip1^ Isoform Pattern after HDACIs Treatment

**Figure 7:**
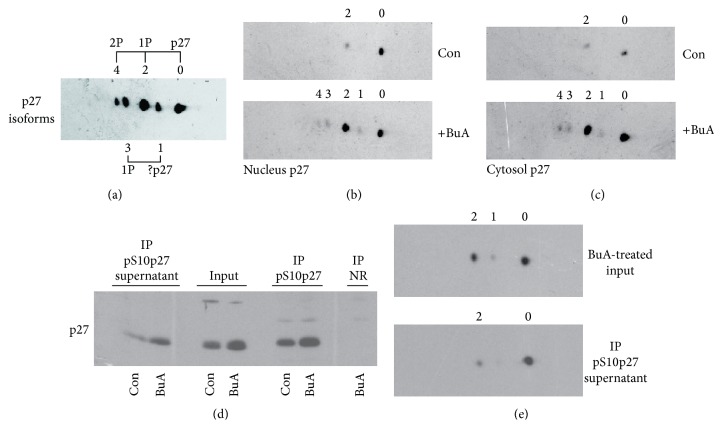
Effect of BuA on the various p27^Kip1^ isoforms. (a) EPN cells were treated with BuA 1 mM for 24 hours. Then, the nuclear extract was prepared and analyzed by bidimensional SDS PAGE and the isoforms were identified by MAb against p27^Kip1^ [25, 26]. Form 0 corresponds to the wild type form and isoform 2 corresponds to the protein with 1 phosphate and isoform 4 corresponds to the CKI with 2 phosphate groups. Form 1 has not been identified and form 3 is the monophosphorylated derivative of form 1. Details on the identification are reported in [25]. Note that the immunoblotting reported corresponds to that which is showed in (b) (BuA-treated nuclear extract) but is exposed to the film for a long time period (i.e., 10 minutes). (b) EPN cells were treated or not for 24 hours with 1 mM BuA. Then, nuclear extracts were prepared and analyzed (1 mg extracts) by bidimensional analysis for p27 isoforms. (c) Exactly as in (b) except that cytosolic extracts were analyzed. (d) EPN cells were treated or not for 24 hours with 1 mM BuA. Then total extracts were prepared and immunoprecipitated with antibodies against phosphoserine 10-p27^Kip1^ (pS10p27). The blot reports the input materials, the IP, the supernatant after pS10p27 removal, and an IP not related (NR). (e) EPN cells were treated for 24 hours with 1 mM BuA. Then cell extracts were prepared and divided into 2 equal aliquots. From one aliquot pS10p27 was removed by IP. Finally, the untreated aliquot and the aliquot from which pS10p27 was removed were analyzed by bidimensional immunoblotting.

One of the most complex features of p27^Kip1^ is its intricate metabolism. Indeed, a series of events, that is, phosphorylation on different amino acid residues and the nuclear/cytosol shuttling, controls its level and function. The complexity of p27^Kip1^ metabolism investigation prompted us to develop a biochemical method based on an initial separation of cell extracts by 2D-SDS PAGE, followed by immunoblotting detection with specific antibodies. We employed this analytical method to characterize in detail the p27^Kip1^ isoforms [25, 26]. We also demonstrated that the p27^Kip1^ bidimensional electrophoretic pattern includes, in addition to the unmodified native form (form 0), three phosphorylated isoforms which contain one (forms 2, 3) or two (form 4) phosphate groups each (Figure 7(a)). Moreover, we identified a p27^Kip1^ isoform (form 1) focalizing between forms 0 and 2. This isoform does not carry a phosphate group but a still unknown postsynthetic modification [27]. The monophosphorylated (form 2) and the wild type (form 0) forms are the most abundant isoforms, while the others are generally scarcely present. Furthermore, the ratio between forms 0 and 2 is strongly dependent on the cellular localization (i.e., nucleus or cytosol) [25, 26]. Finally, isoform 2, which might include multiple p27^Kip1^ isoforms monophosphorylated on different residues, is mainly formed by phosphoserine 10-p27^Kip1^ (pS10p27^Kip1^).

The analysis of p27^Kip1^ isoforms, performed separately on the nuclear and cytosolic fractions from 16-hour-treated EPN cells with or without BuA, is reported in Figures 7(b) and 7(c). We observed that BuA induced in the nucleus the build-up of p27^Kip1^ monophosphoisoform 2. Similarly, in the cytosol, the monophosphorylated isoform increases and biphosphorylated forms appeared. Experiments of immunoprecipitation with anti-pS10p27^Kip1^ antibodies demonstrated a significant increase of this isoform (Figure 7(d)). Moreover, the bidimensional analysis of BuA-treated cell extracts before and after the removal of pS10p27^Kip1^ clearly showed that the increased monophosphorylated isoform is essentially such isoform (Figure 7(e)). This, in turn, suggested that, despite Skp2 decrease, no upregulation of phosphothreonine 187-p27^Kip1^ (pT187p27^Kip1^) occurred confirming the strong BuA-dependent CDK2 activity inhibition.

## 4. Discussion

There is now ample evidence that cancers with low level of p27^Kip1^ or its cytosolic relocalization show a negative prognosis when compared to neoplasias of the same type and stage, but with a higher content of nuclear p27^Kip1^. The finding, initially obtained in colon and breast tumors, has been subsequently confirmed in lung, prostate, gastric, and bladder tumors and other cancers [20, 21, 31, 32, 40]. These burgeoning clinical data, supporting a role for p27^Kip1^ dysregulation in cancer development, suggest that the CKI may be considered a target for therapy. In this context, the results reported here provide support for the use of HDACIs as drugs for increasing p27^Kip1^ levels and might prove to be useful for future anticancer therapies or preventive strategies against human carcinogenesis.

HDACIs represent a class of compounds endowed with significant effects on proliferation, differentiation, and apoptosis. These properties prompted their use in the treatment of a number of pathologies, including cancer, erythropoietic diseases, and, more recently, inflammatory and metabolic pathologies. Besides VPA, which has been for many years the only HDACI introduced in therapy (mainly in the treatment of epilepsy and bipolar disorder and prevention of migraine headaches), vorinostat and romidepsin have been recently approved by the FDA for the treatment of CTCL. Very recently also belinostat received approval for use in therapy of peripheral T cell lymphoma. Hundreds of clinical trials are investigating the clinical use of BuA, PBuA, SAHA, apicidin, MS-275, and other HDAC inhibitors for the treatment of various human malignancies, both as single agents and in combination chemotherapeutic protocols [7, 41–45].

The molecular characterization of HDACI mechanism(s) of action is a major goal of studies on these molecules in view of identifying novel targets for therapy. It is generally accepted that HDACIs effects are mostly due to their capability of reprogramming gene transcription by enhancing the acetylation of either histones or nonhistone proteins, including p53, GATA-1, FOXO proteins, and estrogen and androgen receptors ([7, and references therein], [46–48]). However, the definite identification of HDACIs targets is still lacking.

We have focused our attention on the HDACI effects on p27^Kip1^, since this Cip/Kip inhibitor plays a critical role in malignant transformation [20, 21, 40, 49–53] and, thus, its handling might contribute to explain the putative antitumoral HDACIs activities.

p27^Kip1^ is a critical regulator of cell division cycle, since it interacts with and inhibits several cyclin/CDK complexes. The protein also modulates, in mid-G1, the assembly and nuclear import of cyclin Ds-CDK4/6 complexes. p27^Kip1^ cellular content is regulated by intricate posttranslational mechanisms and by proteasome removal ([20, 21] and references therein). p27^Kip1^ degradation is controlled by at least two distinct ubiquitination processes which occur separately in the nuclear and in the cytosolic compartments. Nuclear ubiquitin-dependent p27^Kip1^ degradation requires an initial phosphorylation on T187, catalyzed by active CDK2 in G1 → S phase transition [51, 52]. T187 phosphorylation permits p27^Kip1^ recognition by Skp2, the recognition subunit of the E3 ligase of SCF-type complex, formed by Skp2 itself, together with Cks1, Skp1, Cul1, and Roc1 [53, 54]. Conversely, cytosolic ubiquitination occurs in a manner independent of T187 phosphorylation and is required for the G1 reentry of cells from G0 phase [55, 56]. The E3 complex responsible for the cytosolic removal (named KPC) has been identified and characterized [57, 58]. Ubiquitinated p27^Kip1^ is degraded by proteasome activities both in the nucleus and in the cytosol. Additional degradation mechanisms have been putatively identified in transgenic mice harboring p27^Kip1^ mutated gene [59].

We found that different HDACIs increase, in a number of cellular models, p27^Kip1^ abundance. This effect, in spite of the frequent HDACI capability to positively modulate gene expression, is not due to an upregulation of* CDKN1B* transcription. We also observed that p27^Kip1^ increase occurs in both nuclear and cytosolic compartments, allowing the exclusion of protein relocalization as the cause of the CKI accumulation.

When we investigated p27^Kip1^ turnover, we observed a stabilization of the protein in BuA-treated cells, suggesting that at least one step of the CKI degradation pathway is downregulated by HDACIs. Then, we ruled out the possibility that BuA acts as a general inhibitor of ubiquitination activity (Figure 4(c)), while we observed a strong HDACI-related downregulation of Skp2, an essential component of p27^Kip1^ ubiquitination complex.

Various experiments demonstrate that Skp2 decrease occurs by using different HDACIs and in several distinct cellular models. Such data appear of importance, in that the effect might be considered as a frequent response to these drugs, independently of the specific cellular context. Our results confirm previous data obtained employing specific HDACIs and distinct experimental models [29–31]. In one cellular model, that is, UT-7 cells, BuA does not downregulate Skp2. Preliminary data suggested to us that HDACIs also do not inhibit the proliferation of these cells (not reported). Future studies are necessary for clarifying the lack of effect in this cellular model.

Although we did not identify definitely the mechanism(s) by which HDACIs decrease Skp2 protein, we initially observed that these compounds reduce Skp2 mRNA. However, Skp2 transcript reduction was statistically significant only after 24 hours of treatment while it might not be detected after 48 hours of BuA addition. On the other hand, Skp2 protein reduction appears quantitatively more significant and precocious than that of the transcript, since it was statistically detectable at 16 hours of treatment. In addition, at 48 hours of treatment very scarce Skp2 protein can be found by immunoblotting. These results indicated the occurrence of nontranscriptional mechanisms leading to BuA-dependent Skp2 decrease.

It has been demonstrated that Skp2 mRNA/protein level are very low in G0 and early G1 [60]. Since HDACIs induce a rapid accumulation of cells in G1 phase (Figure 1(d)), we initially hypothesized that Skp2 downregulation is a direct consequence of G1 phase arrest. However, cytofluorimetric studies of BuA-, PBuA-, and VPA-treated K562 cells show a very rapid build-up of the cells in G1, corresponding to a fall-down of cells in S phase (a cell cycle phase in which Skp2 abundance is at its maximum) persisting up to 24 hours; afterwards, among 24 and 48 hours of incubation, the S phase cell number rises at higher percentage while G1 phase cells decrease. These data suggest the occurrence of a complex effect on the transitions between G1 → S and S → G2/M.

The FACS kinetics is clearly discordant with the analysis of Skp2 protein cellular content. Indeed, Skp2 continued to decrease and it almost disappeared at 48 hours of incubation with BuA. These findings again suggest to us that if an effect of cell cycle synchronization might influence Skp2 abundance, it is not the only phenomenon at the basis of Skp2 protein level modulation and that multiple mechanisms (translational and posttranslational) controlling Skp2 level are modulated by HDACIs.

As shown in Figure 4(a), the treatment with proteasome inhibitor slightly increases Skp2. Analogously, the contemporaneous addition of BuA and ALLN reduces the effect of BuA alone. Both these findings suggest, in agreement with data in literature, that Skp2 levels are regulated, at least in part, by proteasome degradation. As for p27^Kip1^ and many other proteins, Skp2 posttranslational modifications might drive its metabolism. Skp2 stability is regulated by acetylation. Particularly, the protein has been reported to be acetylated by p300 on K68 and K71, therefore becoming more stable [61]. Analogously, inhibition of SIRT3 increases Skp2 acetylation, increasing the protein through impairment of its proteolysis [61]. However, NAM (a powerful inhibitor of SIRT3) does not affect Skp2 and p27^Kip1^ abundance. Thus, acetylation should be discarded as a mechanism for regulating p27^Kip1^ levels.

A further mechanism regulating Skp2 degradation is its phosphorylation on S72 catalyzed by Akt/PKB [35, 36] or on S64 due to active CDK2/CDK1 [37]. Phosphorylation on S72 induces two major molecular effects, that is, the relocalization of Skp2 in the cytosol (and its binding to 14-3-3 protein) and a putative increased formation of SCF complex [35, 36]. In turn, the relocalization in the cytosol might stabilize the protein [35, 36]. Although we confirmed that HDACI might inhibit Akt1 and thus might reduce the phosphorylation of Skp2 on S72, we did not evidence any remarkable change in the localization of Skp2 during the initial 24 hours of treatment. CDK2/CDK1-dependent Skp2 phosphorylation on S64 also stabilizes the protein hampering its interaction with APC-Cdh1 (which targets Skp2 for degradation). Thus, the strong reduction of CDK2 activity due to BuA might contribute to Skp2 reduction. However, additional experiments are required for substantiating this hypothesis and for analyzing the role of other putative Skp2-phosphorylating kinases.

The importance of Skp2 downregulation by HDACIs is not confined to its role in p27^Kip1^ metabolism. Indeed, the increase of Skp2 content has been reported as an independent marker of cancer malignity and aggressiveness. In particular, Skp2 upregulation has been demonstrated in breast carcinoma, non-small cell lung cancer, colorectal carcinoma, gastric carcinoma, renal cell carcinoma, early esophageal squamous cell carcinoma, malignant melanoma, prostate cancer, chronic myelogenous leukemia, malignant lymphoma, and several other human cancers [62, 63]. Thus, the discovery that HDACIs downregulate Skp2 levels has important pharmacological implications in cancer development and prognosis. Skp2, as a part of the SCF-Skp2 E3 ubiquitination complex, is required for the removal of several key proteins as Myc, FOXO1, cancer-derived Smad 4 mutants, ISG15 isopeptidase UBP43, and other proteins. Therefore, the protein downregulation should also have profound effects on cell phenotypes and might be important in explaining the numerous additional activities of HDACIs. In this context, it is important to note that up to now there are scarce data on the effect of HDACIs (i.e., BuA and VPA) on the ubiquitination machinery [64]. It has been reported that the HDACIs downregulate HDAC2 content by inducing the expression of E2 ubiquitin conjugase Ubc8 and, contemporaneously, decreasing the level of the E3 ubiquitin ligase RLIM, two components of the complex responsible for the ubiquitination of HDAC2 [64].

In conclusion, our study reports that HDACIs increase p27^Kip1^ content by elongating its half-life. We also show that the effect is due, at least in part, to a reduced CKI degradation through a strong Skp2 downregulation. Finally, the decrease of Skp2 abundance, due to the HDACIs treatment, appears to be due to the convergence of translational and posttranslational mechanisms. Due to the importance of HDACIs in new therapeutic strategies, our data might contribute to unravelling their mechanism(s) of action, identifying p27^Kip1^ as a major molecular target. Moreover, we demonstrate that these molecules might modulate the cellular protein profiles controlling the activity of pivotal ubiquitination engines including SCF-Skp2. This finding appears of importance in the evaluation of the complex molecular effects of these drugs.
